# Anticonvulsant vs. Proconvulsant Effect of *in situ* Deep Brain Stimulation at the Epileptogenic Focus

**DOI:** 10.3389/fnsys.2021.607450

**Published:** 2021-08-02

**Authors:** Ping Chou, Chung-Chin Kuo

**Affiliations:** ^1^Institute of Physiology, National Taiwan University College of Medicine, Taipei, Taiwan; ^2^Department of Neurology, National Taiwan University Hospital, Taipei, Taiwan

**Keywords:** *in situ* DBS, direct current (DC), kindling, seizure focus, mirror focus

## Abstract

Since deep brain stimulation (DBS) at the epileptogenic focus (*in situ*) denotes long-term repetitive stimulation of the potentially epileptogenic structures, such as the amygdala, the hippocampus, and the cerebral cortex, a kindling effect and aggravation of seizures may happen and complicate the clinical condition. It is, thus, highly desirable to work out a protocol with an evident quenching (anticonvulsant) effect but free of concomitant proconvulsant side effects. We found that in the basolateral amygdala (BLA), an extremely wide range of pulsatile stimulation protocols eventually leads to the kindling effect. Only protocols with a pulse frequency of ≤1 Hz or a direct current (DC), with all of the other parameters unchanged, could never kindle the animal. On the other hand, the aforementioned DC stimulation (DCS), even a pulse as short as 10 s given 5 min before the kindling stimuli or a pulse given even to the contralateral BLA, is very effective against epileptogenicity and ictogenicity. Behavioral, electrophysiological, and histological findings consistently demonstrate success in seizure quenching or suppression as well as in the safety of the specific DBS protocol (e.g., no apparent brain damage by repeated sessions of stimulation applied to the BLA for 1 month). We conclude that *in situ* DCS, with a novel and rational design of the stimulation protocol composed of a very low (∼3% or 10 s/5 min) duty cycle and assuredly devoid of the potential of kindling, may make a successful antiepileptic therapy with adequate safety in terms of little epileptogenic adverse events and tissue damage.

## Introduction

Despite the introduction of quite a few new anticonvulsants in the past 20 years, there is a significant proportion of patients who remain refractory to medical treatment and need invasive or interventional therapies. For example, surgical resection of the brain tissues containing the epileptogenic foci may be considered, if all of the foci are mapped to one region whose function could presumably be compensated for by the other areas. Anterior temporal lobectomy (including amygdalohippocampectomy), thus, has been a prevalent practice for refractory temporal lobe epilepsy; however, the cure rate is not high (de Tisi et al., 2011; Bonelli et al., 2013; Hequette et al., 2013; Boucher et al., 2015), and adverse events, such as memory impairment, hemorrhage, and personality changes are by no means negligible (Gleissner et al., 1998; Sabsevitz et al., 2001; Lee et al., 2002; Jonker et al., 2018). On the other hand, vagus nerve stimulation (VNS) and deep brain stimulation (DBS) of the anterior thalamus are two broad-sense neural stimulation therapeutic maneuvers for refractory seizures. Although less invasive than surgical resection, these stimulation procedures are subject to problems, such as low efficacy and high incidence of adverse events, including cough, hoarseness, difficulty in swallowing for VNS, memory impairment, and depression for thalamic DBS (Ramsay et al., 1994; Ben-Menachem, 2001; Hamani et al., 2010; Hartikainen et al., 2014; Sprengers et al., 2017), although restoration of the kindling-induced memory impairment was observed by DBS at the hippocampus (Ghafouri et al., 2016; Esmaeilpour et al., 2017).

With the same idea as precision medicine, DBS right at the epileptogenic focus (*in situ* DBS) could be a feasible and more promising maneuver against refractory seizures because of its non-destructive, reversible, and adjustable nature. Also, it is technically more feasible in terms of the less strict requirement for a clear-cut demarcation of the seizure foci. Traditionally, high-frequency stimulation (HFS) (>70 Hz) was applied for the possibility of depolarization of the neurons and thus inhibition (inactivation) of the Na^+^ and Ca^2+^ channels (Beurrier et al., 2001). Decrease and/or desynchronization of neuronal discharges may ensue to decrease neurotransmitter release and summation of excitatory postsynaptic potential, and consequently the relevant neural plasticity (Shen et al., 2003; Kim et al., 2012; Guo et al., 2018; Chang, 2018). Low-frequency stimulation (LFS) (<20 Hz) has also been tested for the possibilities of minimization of tissue damage (Weiss et al., 1995; Goodman et al., 2005; Wu et al., 2008; Kile et al., 2010; Jalilifar et al., 2017) and induction of long-lasting hyperpolarization, although the mechanistic rationales are even less clear (Jahanshahi et al., 2009; Rashid et al., 2012; Ghotbedin et al., 2013; Namvar et al., 2017; Paschen et al., 2020). In addition to the uncertainties in the mechanism of action, the apparent efficacy of pulsatile DBS (either HFS or LFS) is highly variable as the ideal pulse protocol has remained to be settled (Rajdev et al., 2011; Sobayo and Mogul, 2016; Zhang et al., 2019). In this regard, a rarely investigated local direct current stimulation (DCS) seems to be more effective than pulsatile stimulation in terms of seizure reduction, but safety issues may have precluded its application (Gaito, 1981; Weiss et al., 1998; Mina et al., 2015).

Kindling is a common phenomenon implicating the establishment of epileptogenicity by repetitive stimulation of the amygdala, the hippocampus, the cerebral cortex, and the telencephalic structures where epileptic seizures almost exclusively originate (Goddard, 1967; Racine, 1972, 1975; Sato, 1982; McIntyre et al., 1993, 1999; Bertram et al., 2008). Since *in situ* DBS must denote long-term stimulation of these potentially epileptogenic structures, it is a concern that a kindling effect and aggravation rather than alleviation of seizures may happen, either manifestly or inconspicuously. We, therefore, explored the scope of stimulation protocols that are capable of a kindling effect and endeavored to antagonize the happening of seizures with an *in situ* stimulation protocol out of the range of electrical kindling (i.e., a prominent “quenching” effect without a side effect of kindling). We found that in the basolateral amygdala (BLA), an extremely wide range of pulsatile stimulation protocols eventually leads to successful kindling. Only protocols with a pulse frequency of ≤1 Hz or a DC would never kindle rats. Furthermore, a short (10 s) pulse of DC currents given 5 min before the challenge stimuli or even given to the contralateral side shows a marked suppressive effect on kindling. In the meanwhile, no apparent brain injury was found with the specifically designed DCS protocols. We conclude that *in situ* DCS may make an effective antiepileptic therapy with little epileptogenic adverse events and tissue damage.

## Materials and Methods

### Animal

Eight-week aged male Wistar rats were purchased from BioLASCO Taiwan Co., Ltd., Taiwan, and housed individually in the 12–12 dark/light-cycle controlled environment with free access to food and water in the National Taiwan University College of Medicine, Laboratory Animal Center. The use and care of animals were conducted in strict compliance with the Institutional Animal Care and Use Committee (IACUC, Approval Number: 20130443 and 20160391) and the Association for Assessment and Accreditation of Laboratory Animal Care (AAALAC) guidelines.

### Stereotaxic Surgery

During stereotaxic surgery, rats were anesthetized under a normal saline mixture solution containing 50 mg/ml Zoletil 50 (Virbac, Carros, France) and 10 mg/ml Xylazine (Sigma-Aldrich, MO, United States) for 1 ml/kg *via* intraperitoneal injection and mounted on a stereotaxic frame (NARISHIGE International, Tokyo, Japan). Bipolar insulated tungsten electrodes (0.002 inches in diameter, <0.5 mm apart, California fine wire) were implanted into target brain regions for electrophysiological recording. Locations which refer to the Paxinos and Watson brain atlas were as follows: the prelimbic cortex (PrL): 3.0 mm AP, 0.75 mm ML, and 0.4 mm DV; the mediodorsal thalamus (MD): –3.0 mm AP, 0.7 mm ML, and 6.0 mm DV; BLA: –3.0 mm AP, 5.0 mm ML, and 8.5 mm DV (relative to bregma). An extra pair of electrodes was buried into the left BLA (L-BLA) for both kindling and bipolar DCS stimulations.

### Kindling Procedures and DCS

**FIGURE 1:**
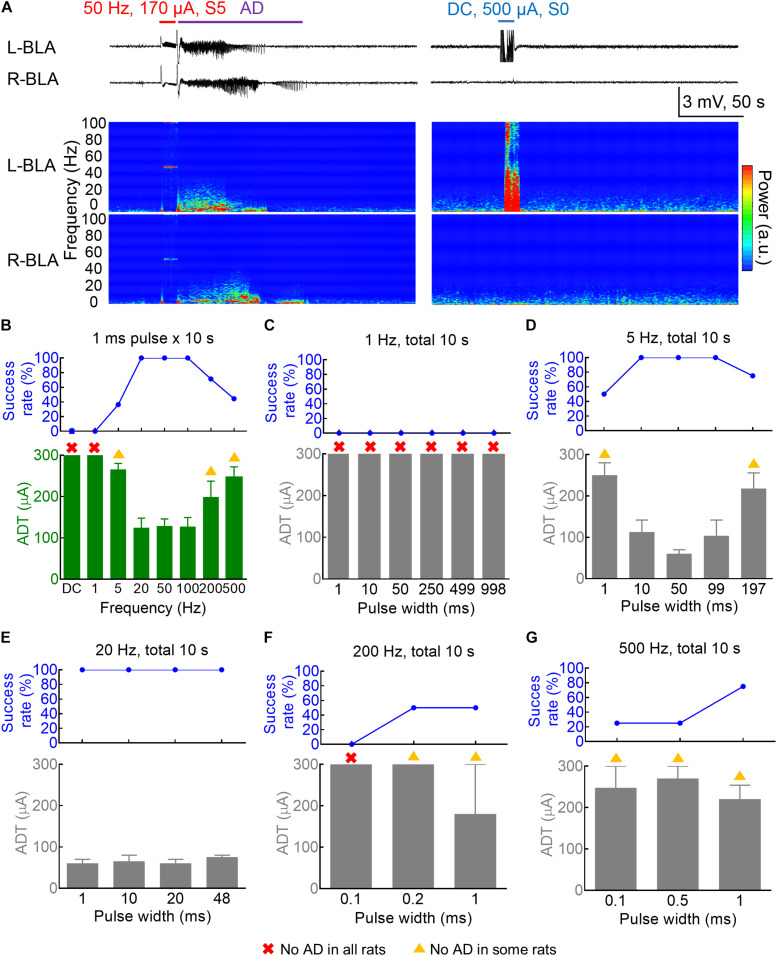
Dependence of the amygdala kindling effect on the pulse frequency and pulse duration. **(A)** Left: The 50 Hz × 10 s (±170 μA) kindling stimuli (red) on the 3rd day readily elicit Racine S5 seizure behavior and afterdischarge (AD) in the bilateral basolateral amygdala (BLA). Samples of local field potential (LFP) (upper panels) and the spectrogram (lower panels) are shown. Right: DC × 10 s (blue) (500 μA, the last session on the 4th day of kindling) elicits no AD or behavioral abnormality. **(B)** The AD threshold (ADT) at the left basolateral amygdala (L-BLA) (lower panel) was measured with different pulse frequencies (*n* = 11, 9, 14, 7, 7, 9 for the 5, 20, 50, 100, 200, and 500 Hz, respectively). The success rate (upper panel) is 100% with 20–100 Hz stimulation, decreases with 5 and 200–500 Hz, and 0 with 1 Hz-pulse or DC stimulation. The red cross denotes that no AD could be elicited in any rats even with the highest currents (300 μA). The yellow triangle denotes that no AD could be elicited in some rats. The axes and symbols are the same in the following: **(C)** No ADT could be defined for the 1 Hz stimulation (*n* = 3) even in different pulse widths 1–998 ms. **(D)** The 5 Hz kindling stimuli are effective with a pulse width of 10–99 ms, but could also elicit AD with shorter and longer pulse width (1 and 197 ms) with lower success rates (*n* = 4). **(E)** With 20 Hz frequency, AD could always be successfully induced with a wide range of pulse width (1–48 ms) (*n* = 2). **(F,G)** High-frequency stimulation (200 and 500 Hz, *n* = 2 and 4) could successfully elicit AD with a pulse width of 1 ms or even < 1 ms.

**FIGURE 2:**
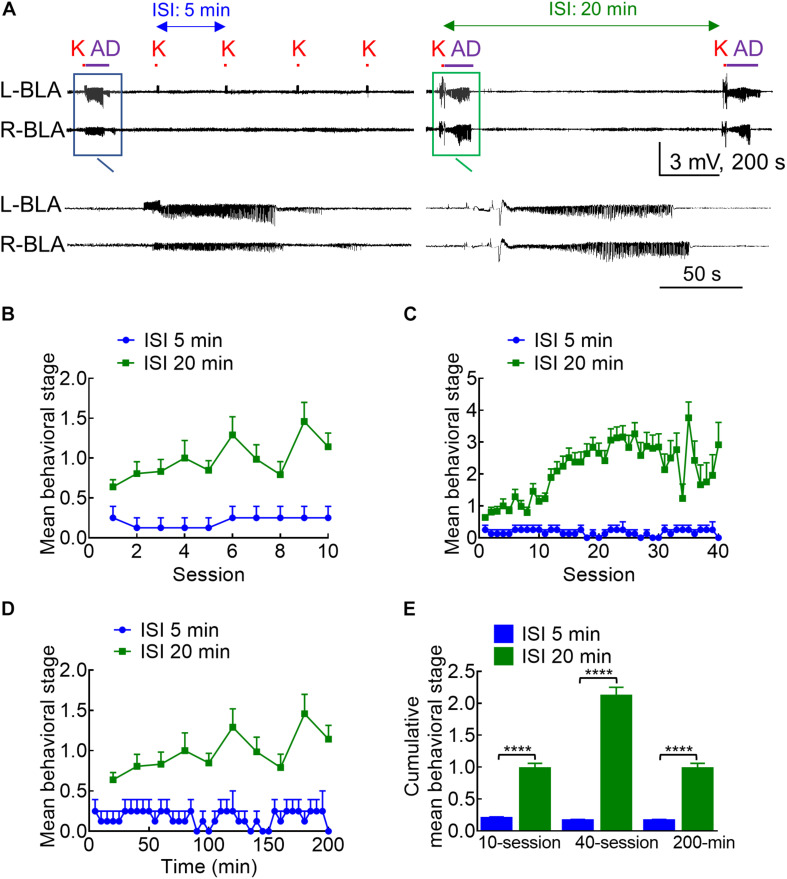
Different kindling effects with different intervals between the 10 s stimulation intersession interval (ISI). **(A)** Samples of LFP were recorded in the BLA with an ISI of 5 min (left panel) or 20 min (right panel). K denotes the 10 s kindling stimulus. Only one episode of AD was induced with 5 min ISI, but nearly every stimulus with 20 min ISI induces AD (a close look is provided in the boxed panels). **(B)** Comparison of the mean behavioral stages for 10 sessions of stimulation between the 5 and 20 min ISI groups shows that the stage is always higher with the latter than with the former. **(C)** The mean behavioral stage for 40 sessions of 20 min ISI (10 sessions/day, completed in 4 days) than the 40 sessions of 5 min ISI (completed in 1 day). **(D)** The mean behavioral stage in a total elapsed time of 200 mins is higher with 20 min ISI (10 stimulation sessions) than 5 min ISI (40 stimulation sessions). **(E)** The cumulative mean behavioral stage is always higher with 20 min ISI than with 5 min ISI, no matter the mean stage is based on 10 sessions (*n* = 10, 10 from the mean of 4 rats for 5 min ISI and the mean of 36 rats for 20 min ISI, respectively), 40 sessions (*n* = 40, 40 from the mean of 4 rats for 5 min ISI and the mean of 36 rats for 20 min ISI, respectively), or 200 min elapsed time (*n* = 40, 10 from the mean of 4 rats for 5 min ISI and the mean of 36 rats for 20 min ISI, respectively). ****p < 0.0001, Mann–Whitney *U* test.

Sessions of 10 s, 50 Hz biphasic square pulses (1 ms each phase) were delivered one time every 20 min (or 5 min) with a stimulus generator (STG4002, Multichannel Systems, Harvard Bioscience, Inc., MA, United States) for 10 sessions/day × 4 days (or when an animal was fully kindled, refer to Section “Electrophysiologic recordings and data analysis”). For the different frequencies of stimulation (e.g., Figure 1), the pulse width was kept at 1 ms unless otherwise specified. If the pulse width was changed, the change was limited to the positive phase. The intensity for kindling stimulation was set at the afterdischarge threshold (ADT), which was the lowest current amplitude capable of induction of epileptiform afterdischarge, determined by the changes of the stimulation currents from 50 to 300 μA in 10 μA increment/2 min Since the ADT in BLA is smaller than in the hippocampus, and on average no larger than 100 μA (Goddard, 1967; McIntyre et al., 1993; Bertram et al., 2008), we chose to have 300 μA as the highest current to avoid inadvertent injuries or other confounding features as much as possible. The afterdischarge was defined as spike-and-wave discharge with a frequency > 1 Hz, an amplitude > 2 times of baseline, and lasting longer than 5 s following the cessation of the stimulation currents. We evaluated behavioral scores at every session of stimulation with modified Racine’s stages: stage (0) normal behavior; (0.5) afterdischarge, absence-like behavior; (1) facial and mouth clonus; (2) head nodding; (3) unilateral forelimb clonus; (4) bilateral forelimb clonus with rearing; (5) rearing and falling; (6) wild running and jumping; and (7) tonic posturing (Sperber et al., 1990). Except for Figure 2, there were in general, 10 sets of 10 s stimuli separated by 20 min for every single day (totally 40 stimuli given in 4 days). The mean of the highest Racine stage reached for the 10 sets of stimulation is defined as the mean behavior stage for each day. In Figure 2, the kindling effect by 40 sets of 10 s stimuli separated by 5 min in 1 day was also explored. The animal is considered as fully kindled when stage 5 seizures (or higher) appeared three times, irrespective of 1 day or separate days (Mohammad-Zadeh et al., 2007; Wu et al., 2008; Grabenstatter and Sutula, 2009). The DCS was applied also in 10 s sessions of 100 μA DC with the kindling stimuli in different chronological sequences with the kindling stimuli (specified in the Figure Legend).

### Electrophysiologic Recordings and Data Analysis

Signals from the target brain regions were recorded *via* a 1400-gain analog amplifier (Model 3600, A-M Systems Inc., WA, United States) with a band-pass filter of 0.3–3 kHz and a notch filter to remove the line frequency noise of 60 Hz. The recordings were then converted into 25k-sampled digital signals *via* a digital-to-analog converter (DataWave Technologies, NJ, United States) and separated through a Butterworth second-order finite impulse response (FIR) filter with 6 dB/oct roll-off to get local field potentials (LFPs, 100 Hz low-pass) afterward.

The signals of LFPs were down-sampled to 500 Hz, and characterized in the frequency domain by power spectrum density (PSD) function in Welch’s method with a 1024-point length and half overlapping Hamming window (Clampfit 10, Molecular Devices, CA, United States). The resolution of PSD was 0.488 Hz in each signal. For the time-frequency analysis (spectrogram), each data point was shifted by 1 s for the computation of PSD until the completion of the full-length signal (SciWorks 10.0, DataWave Technologies, NJ, United States).

Instantaneous phase of LFP recordings was extracted with 21st order FIR in Hamming window and was filtered for different frequency bands *via* Hilbert transform (NeuroExplorer, Nex Technologies, CO, United States). Phase synchronization of two signals was estimated by the correlation of determination (*R*^2^) value, which ranges from 0 to 1, representing low- to high-phase synchronization of the signals. Signals were cut into 1 s epochs as data points and were shifted by one data point to the next epoch to obtain *R*^2^ until the full length was calculated. Values of *R*^2^ higher than 0.5 for constitutive 2 s were considered as significant synchronization.

### Perfusion

Rats were sacrificed with an intraperitoneal injection of Zoletil 50 in overdose and in a passage of 20 s, 5 mA constant current through electrodes to mark the positions. Then, the rats were perfused through the ascending aorta with phosphate-buffered saline (PBS) and 4% formaldehyde. The brain was removed and stored at 4°C in 4% formaldehyde overnight for postfixation, and then moved to 25% (w/v) sucrose solution for cryoprotection. The brain was sliced by a sliding microtome (CM1950, LEICA, Wetzlar, Germany) at 30 μm thickness for cryosection and stained with cresyl violet acetate (Sigma Aldrich, MO, United States) or immunostaining. We analyzed only the electrophysiological data from the rats in which the electrodes were assessed to be in the correct positions.

### Immunostaining

To detect cFos activity, rats were perfused 1 h after the last behavioral seizure. The frozen brain sections were permeabilized with 0.3% PBS with Tween 20 (PBST) and blocked by 0.3% PBST containing 3% fetal bovine serum (FBS) for 2 h. Sections were then incubated with the primary antibodies (rabbit anti-cFos, 1:500 dilution, ABE457, Millipore; mouse anti-GFAP, 1:200 dilution, G3893, Sigma-Aldrich, MO, United States) in blocking buffers overnight at 4°C. The residual antibodies were washed off with 0.1% PBST, and the sections were then incubated with the secondary antibodies (Alexa Fluor 488-conjugated goat-anti-rabbit; Alexa Fluor 555-conjugated goat-anti-mouse, 1:200 dilution, A-11034; A-21422, Invitrogen, MA, United States) and Hoechst (1:1000 dilution, H3570, Invitrogen, MA, United States) in blocking buffers for 2 h in room temperature.

The slides were viewed using an x10 or x20 objective taken stitching panorama or high-resolution *z*-stacks imaging under a fluorescence microscope (AxioImager, Carl Zeiss AG, Oberkochen, Germany) or a confocal microscope (LSM 880, Carl Zeiss AG, Oberkochen, Germany), respectively. Images were processed by Zen software (Carl Zeiss AG, Oberkochen, Germany) with contrast adjustment and intensity maximization in the confocal *z*-axis. Signal density was measured from ImageJ (National Institute of Health, MD, United States), the size of immunoreactive cells was calculated relative to the subthreshold background in the selective area.

### Behavioral Test

Open-field tests were used to evaluate the locomotion activities of rats. A bipolar electrode was implanted into L-BLA to conduct DCS while rats were free-moving in a black acrylic box with the arena ranged in 44 × 44 cm and the motor activities were recorded *via* a hanging webcam (C270, Logitech, Lausanne, Switzerland) in 30 frames per second (fps). Each trial was 5 min in length and was real-time analyzed by tracking software (EthoVision XT, Noldus Information Technology, Wageningen, Netherlands).

### Statistics

Grouped data are reported as averages ± SEM. All statistical tests are two-sided. For the comparison between two individual groups, Mann–Whitney *U* test was applied. Otherwise, the Wilcoxon matched-pairs signed-rank test was applied in the paired groups (Prism 6, GraphPad Software Inc., CA, United States).

## Results

### Amygdala Kindling Threshold Could Be Achieved With a Very Wide Frequency and Pulse Width Range of Stimulation

We first investigated the frequency-dependence of the kindling stimuli with the standard pulse width and current polarity “afterdischarge (AD)” of kindling. Figures 1A,B show that the “threshold” current amplitude necessary for the induction of AD (ADT) roughly is the same low for stimuli given in 20–100 Hz and the success rate (the percentage of rats with AD) is very high (100%). Stimuli given in 5 or 500 Hz could still elicit AD, although the threshold current amplitude necessary for AD is higher, and the success rate is lower. On the other hand, no AD or seizures could ever be elicited with a 10 s DCS even with a 500 μA current (Figure 1A and Supplementary Figure 1). It seems that a pulsatile stimulation is necessary for the induction of AD and successful kindling or epileptogenesis. The frequency range of the pulsatile stimulation for effective kindling, however, is very wide, at least from 5 to 500 Hz. The spectrograms in Figure 1A show that the major oscillations are under ∼10 Hz. We, therefore, would focus on this ≤ 10 Hz frequency band for further characterization of brain oscillations in the following sections of the study. We further explored more characteristics of effective kindling stimulation. About 1 Hz stimulation hardly led to any AD, no matter how long the pulse width is (1–998 ms, Figure 1C). The ADT with 5 Hz stimulation is rather small with a pulse width of 10–99 ms but gets higher with a pulse width of 197 ms (Figure 1D and Supplementary Figure 1). The success rate to generate AD is 100% in all rats with a pulse width of 10–99 ms and 50–75% with a pulse width of 1 or 197 ms. These findings demonstrate that the AD of kindling at the amygdala could be achieved with not only a very wide range of stimulation frequency but also with a total stimulation time as short as 5 ms per seconds. The success rate of AD induction is 100% for 20 Hz stimulation, with pulse duration as short as 1 ms or as long as 48 ms (Figure 1E). Even with a 500 Hz stimulation, one may still have a chance of AD with pulse duration as short as 0.1 ms or as long as 1 ms (Figures 1F,G).

### The Epileptogenic Effect Could Be Much Weaker With More Frequent Stimulation Sessions

With the characterization of the stimulation pattern within each stimulation session, we turned to the features of the intersession intervals. Counterintuitively, the kindling effect and the behavioral manifestations of seizures are much weaker if the intersession interval (ISI) is deliberately shortened to 5 min (Figure 2, note that the standard ISI is 20 min for all of the other figures in this study). This is consistent with the idea that the kindling stimulation should be given in 10 s sessions, 10 sessions per day with an intersession interval ideally above 20 min (Morales et al., 2014). In any case, this finding sustains that the process of epileptogenesis could be effectively interrupted with a predefined stimulation session given intermittently (e.g., one time in few minutes).

### There Is an Anti-ictogenic Effect of *in situ* DCS If Given With or Immediately After the Challenge Stimuli

**FIGURE 3:**
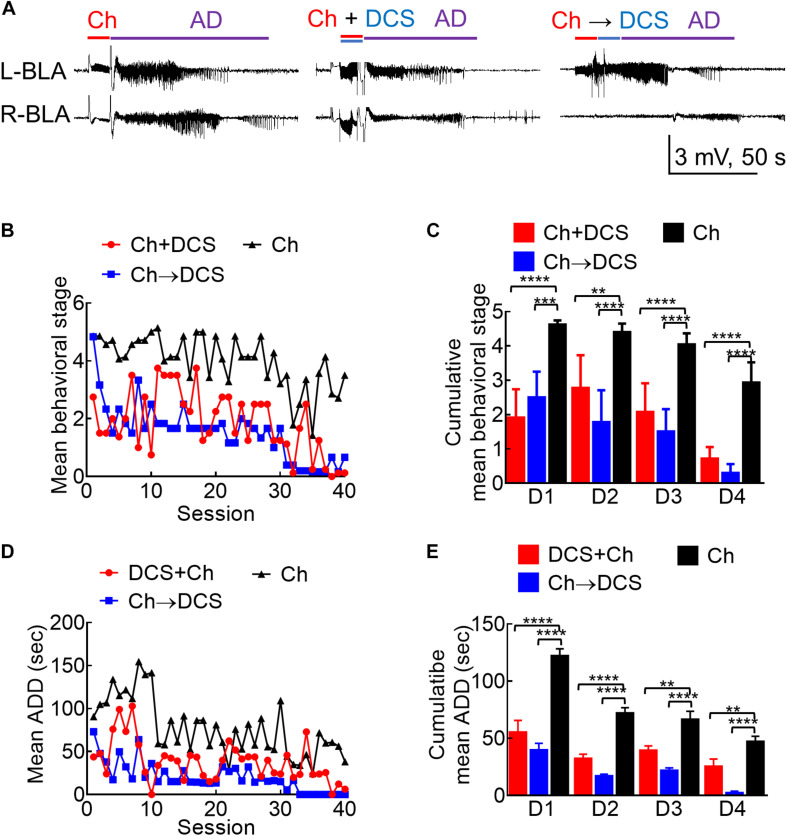
The anticonvulsant effect of DCS given with or immediately after the challenge stimuli in fully kindled rats. **(A)** Sample LFP recordings (left panel) show AD elicited with a challenge stimulus in the left basolateral amygdala (L-BLA) in a fully kindled rat. Behaviorally, this would usually cause Racine stage 5 seizures as in this case, 100 μA, 10 s *in situ* direct current stimulation (DCS) was given concomitantly with (DCS + Ch, middle panel) or immediately after (Ch→DCS, right panel) the challenge stimulus (Ch) in the fully kindled rat. The values of AD are slightly shortened in both DCS conditions (with behavioral stages attenuated to stage 3 and stage 4, respectively). **(B)** The mean behavioral stage at each of the 40 challenge stimulation sessions with DCS given concomitantly with or immediately after the challenge (Ch + DCS, red symbols, *n* = 4; Ch→DCS, blue symbols, *n* = 6) is in general, lower than that with challenge only (Ch, black symbols, *n* = 7). Only the mean values are shown [SEM is omitted for the sake of clarity]. **(C)** The cumulative mean behavioral stage in each of the 4 days is lower in both DCS groups if compared to the challenge only group (*n* = 10, 10, 10 each day from the mean of 4, 6, 7 rats for Ch + DCS, Ch→DCS, and Ch group, respectively). **(D)** The mean AD duration (ADD) from L-BLA after each challenge stimulation is shortened by DCS given with or after the challenge (DCS + Ch, red symbols, *n* = 4; Ch→DCS, blue symbols, *n* = 5; Ch, black symbols, *n* = 3). **(E)** The cumulative mean ADD in each of the 4 days is lower in both DCS groups if compared to the challenge only group (*n* = 10, 10, 10 each day from the mean of 4, 5, 3 rats for Ch + DCS, Ch→DCS, and Ch group, respectively). *p < 0.05; **p < 0.01; ***p < 0.001; ****p < 0.0001, Mann–Whitney *U* test.

Based on the findings from Figures 1, 2, it is evident that a wide range of the amygdala stimulation could be epileptogenic, and an epileptogenic or relevant adverse effect and anti-epileptogenic effect could well be intermixed. In this regard, it is of note that stimuli repeated ≤ 1 Hz or DC are clearly devoid of the kindling or proconvulsant effect (Figures 1B,C). Accordingly, LFS (≤1 Hz) has been shown to have a prominent anticonvulsant effect. We, therefore, chose to study the antiepileptogenic and anti-ictogenic effects of DBS with DC in detail with different sequences of stimulation. A session of 10 s DCS for at least 5–20 min apart from the next stimulation could also be viewed as an extremely LFS (i.e., ∼0.03—0.008 Hz). The 10 s DC with 100 μA amplitude is chosen as it is roughly the 50% effective dose for the inhibition of AD if given with the kindling or challenge stimuli (Supplementary Figure 2). Besides, there is no discernible change in behaviors including moving distance and rearings (which may be related to the anxiety level; Koek et al., 2014; Sturman et al., 2018; Schönfeld and Wojtecki, 2019) with the 100 μA DCS (Supplementary Figure 3). The anti-ictogenic effect was tested first in fully kindled rats, as a fully established seizure focus is the most common clinical scenario. In a fully kindled rat, behavioral seizures of Racine stage 4 or 5 could always be induced if a 10 s challenge stimulation with a current amplitude of the ADT is applied to the epileptogenic focus. Interestingly, if DC currents are applied together with or after the challenge stimulation in a new fully kindled rat, the AD and behavioral seizures are markedly attenuated (Figures 3A–E). If one follows the stimulation pattern of kindling acquisition (e.g., 10 sessions of 10 s challenge stimulation every 20 min per day for 4 days), it is evident that the anti-ictogenic effect gets better and better from day 1 to day 4 of DCS (Figures 3C,E). Even the ictogenic effect of the challenge stimulation itself shows a tendency of decrease from day 1 to day 4 without DCS, suggesting a mixed ictogenic and anti-ictogenic effects of the challenge stimulation partly resembling the findings in Figure 2 (refer to Section below).

### There Is an Even Stronger Anti-ictogenic and Anti-epileptogenic Effect of *in situ* DCS If Given Before the Challenge Stimuli

**FIGURE 4:**
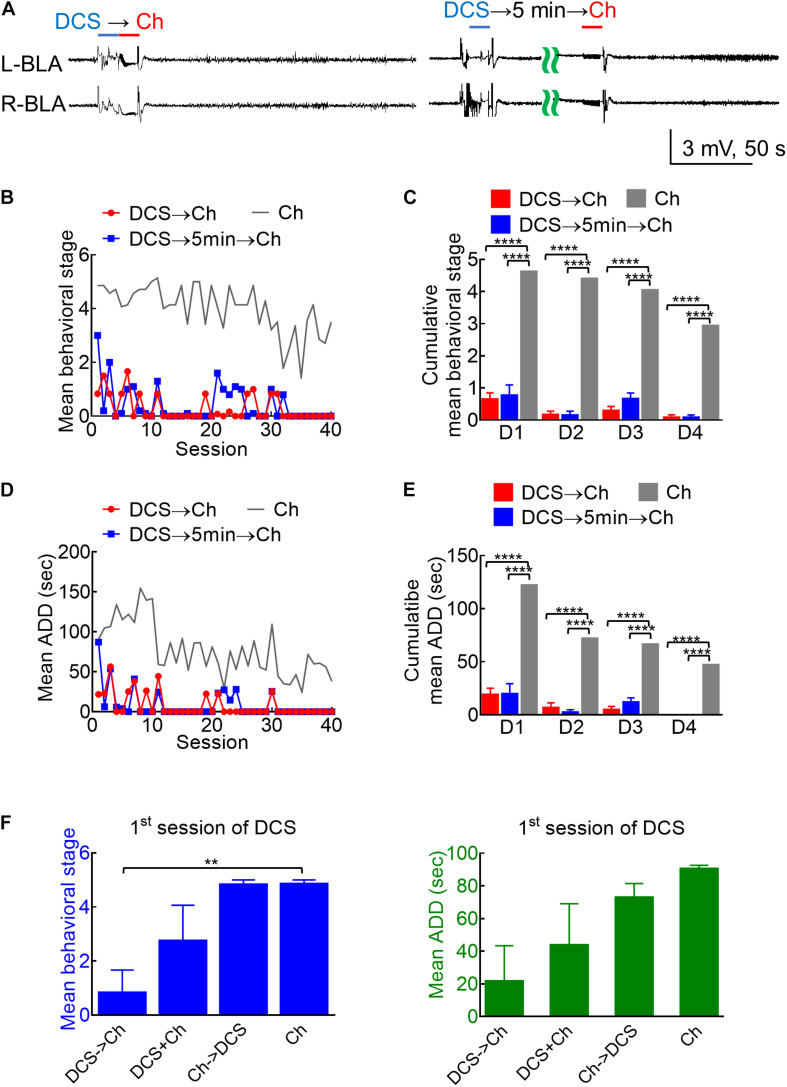
Marked anticonvulsant effect of DCS in fully kindled rats if given immediately before or 5 min before the challenge stimuli. **(A)** Sample LFP shows that 100 μA, 10-s DCS right before (DCS→Ch, left panel) or 5 min before challenge (DCS→5 min→Ch, right panel) markedly suppresses AD in bilateral BLA (and the rat expressed no seizure behaviors). **(B)** The mean behavioral stage during each session of stimulation by DCS given immediately before or 5 min before the challenge stimulus (DCS→Ch, red symbols, *n* = 6; DCS→5 min→Ch, blue symbols, *n* = 5) is markedly lower than that with a challenge only (Ch, gray symbols, *n* = 7). Only the mean values are shown for the sake of clarity. The gray line is the control data taken from Figure 3B and replotted for comparison. **(C)** The cumulative mean behavioral stage in each of the 4 days is lower in both DCS groups if compared to the challenge only group (*n* = 10, 10, 10 each day from the mean of 6, 5, 7 rats for DCS→Ch, DCS→5 min→Ch, and Ch group, respectively). **(D)** The mean ADD from L-BLA after each challenge stimulation is shortened by DCS given before or 5 min before the challenge (DCS→Ch, red symbols, *n* = 4; DCS→5 min→Ch, blue symbols, *n* = 3). The gray line is the control data taken from Figure 3D and replotted. **(E)** The cumulative mean ADD in each of the 4 days is lower in both DCS groups than in the challenge only group (*n* = 10, 10, 10 each day from the mean of 4, 3, 3 rats for DCS→Ch, DCS→5 min→Ch, and Ch group, respectively). **(F)** At the first session of challenge, the mean behavioral stage (left) is lower, and mean ADD (right) is shorter with DCS given immediately before challenge (DCS→Ch) than that given concomitantly with or immediately after challenge. *n* = 6, 4, 6, and 7; or *n* = 4, 4, 5, and 3 for seizure stage or ADD of Ch→DCS, DCS + Ch, Ch→DCS, and Ch groups, respectively. ***p < 0.001; ****p < 0.0001, Mann–Whitney *U* test.

**FIGURE 5:**
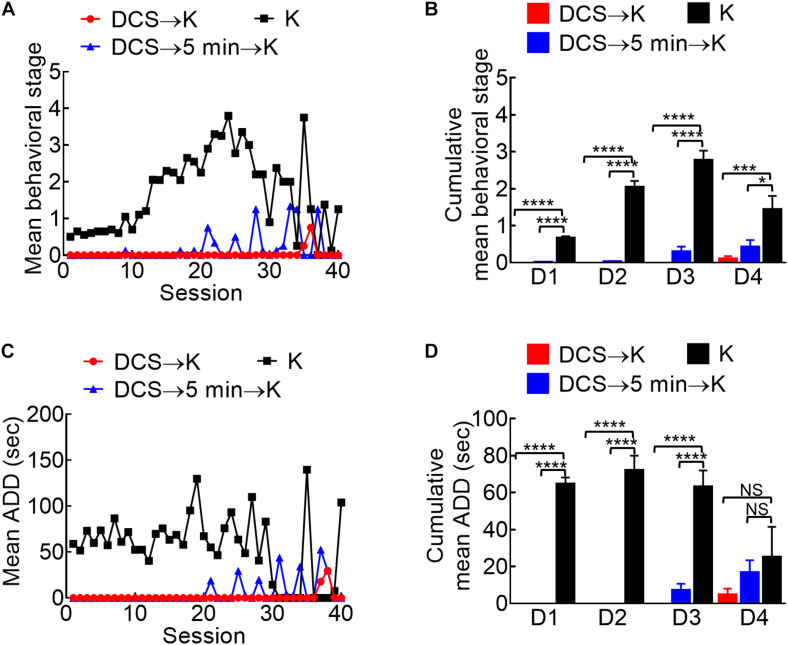
Marked anti-epileptogenic effect of DCS if given either immediately before or 5 min before the kindling stimuli. **(A)** Mean behavioral stage at each of the 40 stimulation sessions with 100 μA, 10-s DCS given immediately before or 5 min before the kindling stimuli (DCS→K, red symbols, *n* = 4; DCS→5 min→K, blue symbols, *n* = 4) is, in general, lower than that with kindling only (K, black symbols, *n* = 10). Only the mean values are shown [SEM is omitted for the sake of clarity]. **(B)** Cumulative mean behavior stage in each of the 4 days is lower in both DCS groups if compared to the challenge only group (*n* = 10, 10, 10 each day from the mean of 4, 4, 10 rats for DCS→K, DCS→5 min→K, and K group, respectively). **(C)** The mean ADD from bilateral BLAs after each kindling stimulation is shortened by DCS given either immediately before or 5 min before kindling (DCS→K, red symbols, n = 3; DCS→5 min→K, blue symbols, *n* = 3; K, black symbols, *n* = 4). **(D)** The cumulative mean ADD in each of the 4 days is lower in both DCS groups than in the kindling only group (*n* = 10, 10, 10 each day from the mean of 3, 3, 4 rats for DCS→K, DCS→5min→K, and K group, respectively). *p < 0.05; **p < 0.01; ***p < 0.001; ****p < 0.0001, Mann–Whitney *U* test.

**FIGURE 6:**
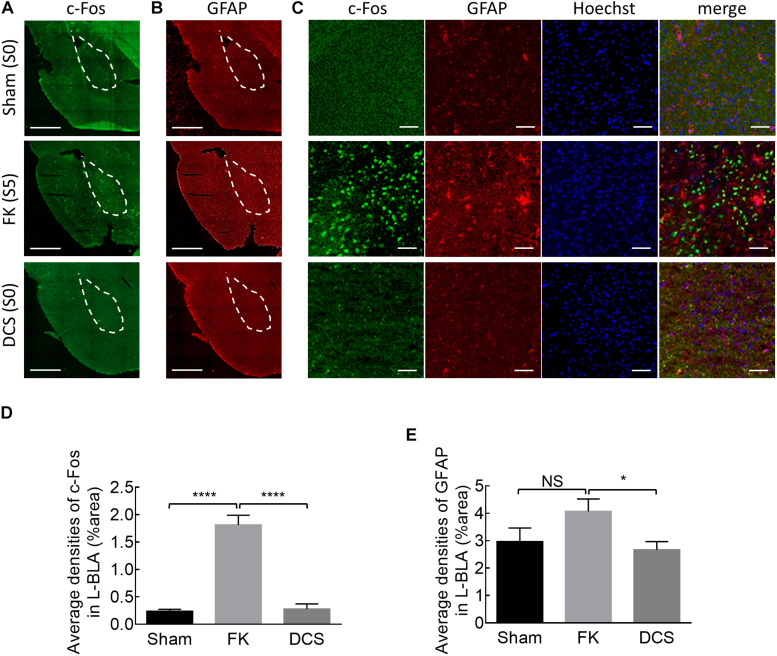
Marked anticonvulsant effect of DCS demonstrated histologically in fully kindled rats. **(A)** Representative immunostaining images show that cFos expression is markedly increased after the rat was fully kindled (FK, middle panel) when compared to the control rat which acts as normal without kindling (sham, upper panel). The cFos expression was attenuated or even is essentially absent in the left basolateral amygdala (L-BLA) (dotted area) after 40 sessions of 100 μA, 10 s DCS applied immediately before the challenge stimuli (DCS→Ch) in a fully kindled rat (DCS, lower panel) if compared to the fully kindled rat without DCS (FK). Behaviorally, the rats were in Racine stage 5 and stage 0, 1 h before being sacrificed in the cases without and with DCS, respectively. **(B)** Reactive gliosis (anti-GFAP) in L-BLA (dotted area) is also markedly increased after fully kindled (FK, middle panel), and reduced with DCS (DCS→Ch, DCS, lower panel) when compared to the fully kindled rat without DCS in the same sections, such as that in Part **(A)**. Scale bars of **(A,B)** are 1 mm. **(C)** The highly magnified and merged images from Part **(A, B)** with cFos (green), GFAP (red), and Hoechst for cell nuclei (blue) staining in L-BLA, show that cFos and GFAP expression is markedly attenuated after DCS. Scale bars are 0.2 mm. **(D,E)** cFos-positive cells and GFAP-positive cells in the L-BLA were quantified and statistically analyzed in sham, FK, and DCS groups. *n* = 10, 22, 16 slices in 3, 4, 4 rats for sham, FK, and DCS groups, respectively. NS*p* > 0.05; *p < 0.05; ****p < 0.0001, Mann–Whitney *U* test.

Given the deleterious rather than enhancing effect on kindling if the stimulation is repeated every 5 min (compared to that repeated every 20 min), we investigated the anti-ictogenic effect of DBS with DC currents at the epileptogenic focus immediately before or 5 min before the challenge stimulus in fully kindled rats (Figure 4A). Both the protocols of DCS show a very strong anti-ictogenic effect, apparently even stronger than that given with or after the challenge stimulus (Figures 4B—E). The much stronger effect of DCS given before the challenge stimuli (Figure 4), and the probable cumulative or carried-over anti-ictogenic effect of the challenge stimulation itself from day 1 to day 4 (Figures 3, 4) implicates that at least part of the anti-ictogenic effect of the DCS may be derived from the action on the subsequent challenge stimuli (i.e., the DCS following a challenge stimulus also serves as the DCS before the next challenge stimuli). We, therefore, try to clarify this issue by examining the effect of DCS on the first challenge stimulus. The anti-ictogenic effect is, indeed, the weakest (even if it is only minimal) if DCS is given immediately after the challenge and the strongest if given before the challenge in this “first challenge” setting (Figure 4F). It is intriguing although the DC itself has no kindling effect, it seems to initiate a process that still lasts for at least quite a few minutes or even longer (refer to section “Discussion”). In addition to the anti-ictogenic effect, the anti-epileptogenic effect of the *in situ* DCS given before the stimulation is examined in Figure 5, where the kindling effect is so attenuated as to be abolished with DCS given immediately or 5 min before each kindling stimulation. Consistently, the cFos expression increased markedly and reactive astrocytes in L-BLA in the fully kindled rats with behavioral seizures are markedly attenuated or abolished by DCS given before the challenge stimuli (Figure 6). The discovery of a therapeutic window up to ∼5 min before the trigger of an established epileptogenic focus carries an important connotation in clinical application. It is thus feasible to gain complete control of epileptic seizures with intermittent and relatively infrequent (e.g., 10 s in 5 min) *in situ* open-loop DBS.

### There Is Also an Anti-epileptogenic and Anti-ictogenic Effect of *in situ* DCS If Given to the Contralateral BLA

**FIGURE 7:**
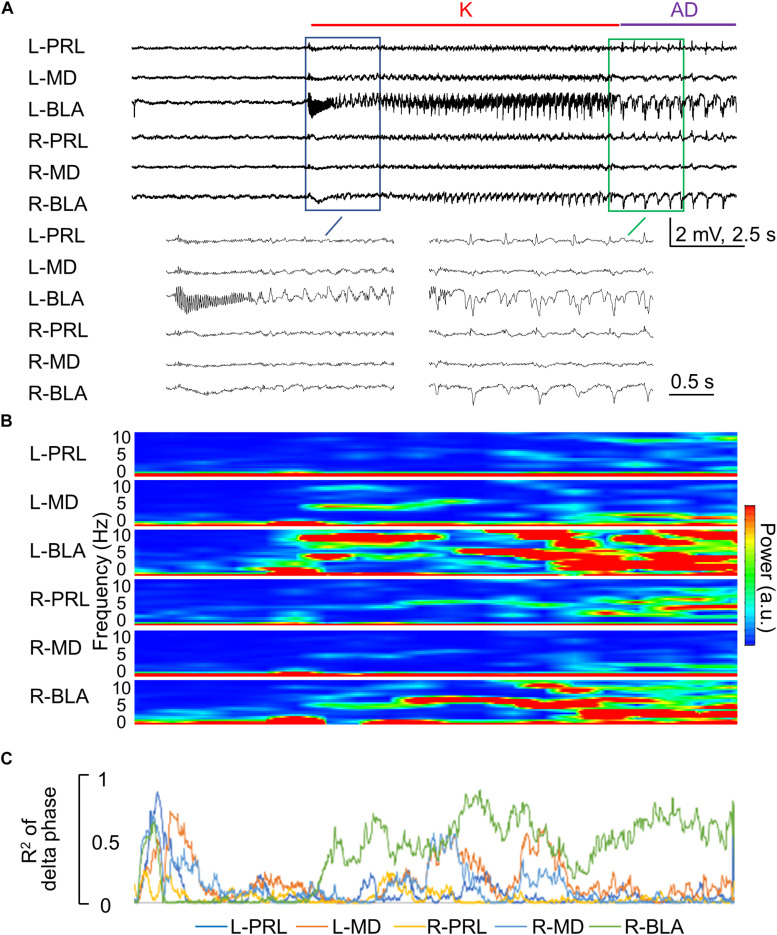
Rapid propagation of epileptiform discharges to the other brain regions during the kindling stimulation. **(A)** Sample LFP recordings show that the epileptiform discharges gradually emerge in different recording sites in the first 1–2 s after initiation and cessation [i.e., AD] of the stimulation in the first session of kindling (enlarged). In this case, the kindling intensity is ±70 μA, and the rats expressed Racine stage 0.5 and 1 behavioral seizures at spike-wave discharge (SWD) and AD, respectively. **(B)** Spectrogram analysis of Part **(A)** shows that the major power of epileptiform discharges is the delta range (1–5 Hz). **(C)** Phase synchronization of delta waves in Part **(A)** between L-BLA and in the different structures shows that the right basolateral amygdala (R-BLA) has by far the strongest synchronization with L-BLA in the 10 s kindling stimulation and immediately afterward, i.e., during the AD. Note that Parts **(B,C)** are analyses based on the recordings in Part **(A)** and thus share the same time scale.

**FIGURE 8:**
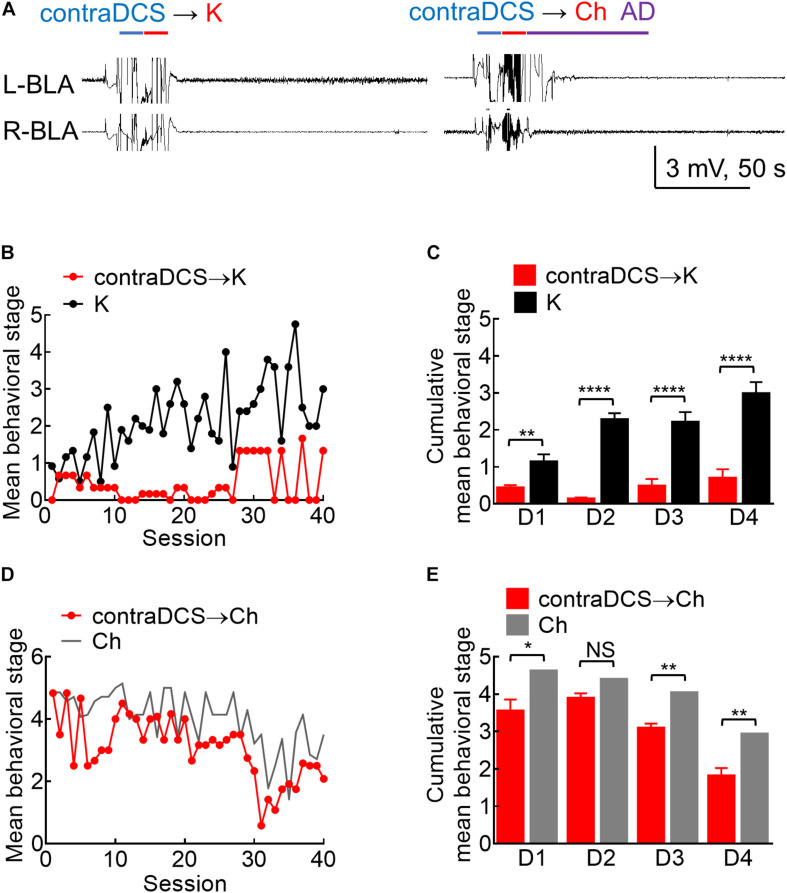
Effects of contralateral DCS if given right before the kindling or the challenge stimuli. **(A)** Sample local field potentials (LFP) show that contralateral 100 μA, 10-s DCS at right basolateral amygdala (R-BLA) right before kindling (contraDCS→K, left) or challenge (contra DCS→Ch, right) markedly suppresses AD (and the rat expressed no seizure behaviors). **(B)** Contralateral 100 μA, 10 s DCS at R-BLA immediately before the kindling stimuli applied to the left BLA (L-BLA) (contraDCS→K, red symbols, *n* = 3; K, black symbols, *n* = 6) markedly lowers the mean behavioral stage when compared to the kindling the only group. Only the mean values are shown [standard error of the mean (SEM) is omitted for the sake of clarity]. **(C)** Cumulative mean behavior seizure stages in each of the 4 days are in general lower with contralateral DCS when compared to the kindling only groups (*n* = 10, 10 each day from the mean of 3, 6 rats for contraDCS→K and K group, respectively). **(D)** Contralateral DCS at R-BLA right before the challenge stimuli in fully kindled rats (contraDCS→Ch, red symbols, *n* = 6) also lowers the mean behavioral stage when compared to the challenge-only group (gray line, data taken from Figure 3B with the symbols omitted). Only the mean values are shown [standard error of the mean (SEM) is omitted for the scale of clarity]. **(E)** Cumulative mean behavior seizure stages in each of the 4 days are in general lower with contralateral DCS when compared to the challenge-only groups (*n* = 10, 10 each day from the mean of 6, 7 rats for contraDCS→Ch and Ch group, respectively). NS*p* ≥ 0.05; *p < 0.05; **p < 0.01; ***p < 0.001; ****p < 0.0001, Mann–Whitney *U* test.

We have shown that the kindling stimulation of L-BLA could readily induce AD bilaterally (Figure 1). The responses between bilateral BLA are especially coherent (Figures 7A–C), demonstrating that BLA kindling is essentially a bilateral response, reasonably analogous to the “mirror” foci of epileptic seizures in clinical settings. We, therefore, investigated the effect of DCS applied to the right BLA (R-BLA) on the kindling and challenge stimulation applied to the L-BLA (Figure 8A). With DCS (DC currents, 10 s) applied right before the kindling stimuli, the acquisition of kindling (a form of epileptogenesis) is strongly inhibited (Figures 8B,C). There is also an anti-ictogenic effect of R-BLA DCS on the effect of challenge stimuli applied to the L-BLA in a fully kindled rat (Figures 8D,E), although the anti-ictogenic effect is again weaker than the anti-epileptogenic effect. These findings indicate that the establishment of epileptogenic foci in BLA or the ictogenesis afterward is, indeed, a bilateral process, which may require an ordered reverberation between both sides. The DCS applied to just one side may therefore have an anti-epileptogenic and anti-ictogenic effect bilaterally. This could carry an intriguing significance in both neurobiology and clinical therapeutic applications (refer to section “Discussion”).

### The Long-Lasting Anti-ictogenic Effect and Long-Term Safety of *in situ* DC Stimulation

**FIGURE 9:**
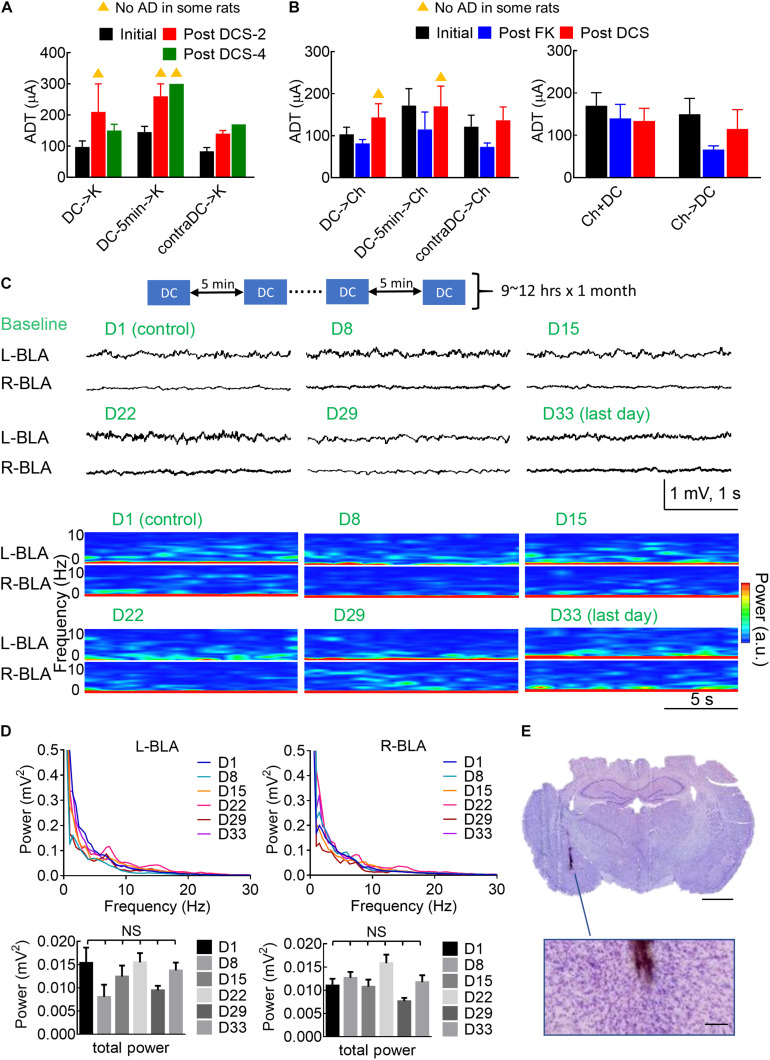
Long-lasting anti-ictogenic effect and long-term safety of DCS. **(A)** The initial ADT denotes the threshold for elicitation of AD. The post DCS-2 and post DCS-4 ADT denote the threshold for the elicitation of AD, 2 and 4 weeks after the initial ADT, respectively, with DCS given immediately before the kindling (DCS→K, *n* = 4) or 5 min before kindling (DCS→5 min→K, *n* = 4), or immediately before kindling to the contralateral side (contraDCS→K, *n* = 3). The yellow triangle denotes that no AD could be elicited in some rats. There is a clear trend that the post-DCS-2 or post-DCS-4, ADT is higher than the initial ADT. **(B)** The post-FK ADT denotes the threshold for elicitation of AD 10 days after the rat was fully kindled. The FK rats then underwent a 4-day challenge stimulation preceded by different forms of DCS analogous to part **(A)** (left: DCS→Ch, *n* = 6, DCS→5 min→Ch, *n* = 5, contraDCS→Ch, *n* = 6; right: Ch + DCS, Ch→DCS, both *n* = 6). The post-DCS ADT denotes the threshold for the elicitation of AD roughly 10 days after the DCS treatment. In this study, is a tendency that the post-DCS ADT is higher than the post-FK ADT if DCS were given before kindling and challenging stimulation. **(C)** Repeated sessions of *in situ* DCS (100 μA × 10 s) were applied to the left basolateral amygdala (L-BLA) every 5 min, 9–12 h a day for 1 month. The baseline electrophysiological activities [LFP recordings and spectrogram analysis] of bilateral BLAs remain rather similar from day 1 to day 33. **(D)** The mean power spectrum densities (PSD, upper) and total power (0.5–100 Hz, lower) of 1 min baseline recordings at bilateral BLAs remain the same from day 1 to day 33 (*n* = 6 each day in 2 rats). NS: p ≥ 0.05, Wilcoxon matched-pairs signed-rank test. **(E)** Histological examination with Nissl stain shows no apparent tissue damage in the vicinity of the electrode (the brown trace). The scale bar is 2 mm (or 0.2 mm for the enlarged figure).

It is interesting to note that *in situ* DCS has given a few minutes before a challenge or a kindling stimulus could have an effect comparable to that given immediately before the stimulus (Figures 4, 5). Figures 9A,B show the possibility of a long-lasting anti-epileptogenic and anti-ictogenic effects in terms of days and even weeks. There is a tendency of elevation in ADT more than 1 or even 3 weeks after DCS (i.e., with no extrinsically applied currents for more than 1 or 3 weeks), no matter the DCS was given immediately or 5 min before the stimuli, or was given contralaterally. This long-lasting effect is consistent with the view that *in situ* DCS, although not epileptogenic or ictogenic by itself, makes a rather long-term change or drives an everlasting ongoing process at the stimulation site (refer to section “Discussion”). In this regard, it is of note that repetitive applications of 10 s DCS every 5 and 20 min for 10 h per day had caused no apparent electrolytic lesion of brain structure after 1 month (Figure 9E). Physiologically, there are also no gross changes in the local field potential recordings or spectrogram analysis (Figures 9C,D). The long-term effect of DCS thus could be inconspicuously by itself, but conspicuously manifest if examined by epileptogenesis and ictogenesis.

## Discussion

### Successful Kindling With a Very Wide Range of Stimuli: Implications on the Mechanism Underlying Epileptogenesis

We have shown that successful kindling or epileptogenesis could be achieved with a very wide range of stimulation protocols applied to BLA (Figures 1A,B; Cain and Corcoran, 1981; Minabe et al., 1986). The basic design of BLA circuitry thus seems to involve self-sustained oscillating activities in response to and outlasting many kinds of extrinsic stimuli; however, there is still a limitation on the stimulation protocols for successful kindling. The stimulation must be given intermittently (with an interstimulus interval of at least a few minutes) and repeated > 1 Hz no matter how long and how strong it is (Figure 1C). In other words, the stimuli have to be pulsatile and repeated at least in the delta range for the triggering of AD. As there is an intrinsic frequency of oscillating discharges in the delta range in either normal or seizure conditions in the BLA circuitry (Chou et al., 2020), we would propose that the trigger stimuli must be harmonics or superharmonics to the intrinsic rhythms of the involved circuitry to be potentially epileptogenic. The DC stimulation, therefore, has no apparent effect on triggering the network activities (Supplementary Figure 4). The other essential attribute is the AD itself, which denotes self-regenerative reverberating network activities outlasting the primary trigger. The reverberating activities, however, are not “stable” but “dissipating” after the trigger, so that timely booster stimuli are required for a cumulative effect toward successful kindling or long-lasting network changes (Goddard et al., 1969; Daigen et al., 1991; Leung et al., 1998; Bertram, 2007; Kandratavicius et al., 2014). Being fundamental attributes of the circuitry, similar processes may also play an imperative role in normal neurocomputation and memory. On the other hand, it is of note that the 10 s set of stimulation should not be repeated too frequently either (Figure 2). This is as if the triggered intrinsic self-regenerative discharges are actively ongoing, additional extrinsic pulses to the network are likely not so in phase and would interfere with the oscillations in progress. It is thus plausible that in a more “materialized” level, there could be structural changes in the network which are brought up by temporospatial specific reverberating activities and may accumulate to contribute to that particular kind of oscillating discharges in return. It would be highly desirable to identify the molecular mechanisms underlying the foregoing structural changes, which may be quite a novel identity because of the unique (“in-phase”) requirement of the stimulation. In this regard, the epileptogenic focus is a network capable of showing orderly reverberating discharges with specific temporospatial sequences of involvement (Chou et al., 2020; Wang et al., 2021).

### Separation of Anticonvulsant From Proconvulsant Effect of Brain Stimulation: The Role and Mechanism of DC Stimulation in the Treatment of Seizures

The kindling or proconvulsant effect of a very wide range of stimulation protocols underscores the importance to define the range of a pure anticonvulsant effect (“quenching without kindling”) when pursuing a successful therapy of seizures with *in situ* DCS. The findings in Figure 2 show that stimuli given in every 5 min markedly attenuate the efficacy of kindling and implicate that the perturbed network could be effectively kindled again only after adequate dissipation of the aroused activities (possibly to avoid the occurrence of out-of-phase extrinsic and intrinsic oscillations, refer to Section Successful Kindling With a Very Wide Range of Stimuli: Implications on the Mechanism Underlying Epileptogenesis). Consistently, we have shown that the kindling effect or epileptogenesis increases in both synchrony and number of the discharging units, and the change in synchrony precedes and outlasts the changes in discharging units and behavioral seizures (Chou et al., 2020). We have also demonstrated in brain slices that after a session of 1 s 60 Hz stimuli, the burst discharges and excitatory postsynaptic events could be increased immediately by nearly ∼10 times when compared to that at the baseline (Wang et al., 2021). The kindling effect, then, slowly dissipates to roughly half of the peak value in ∼45 s. It is thus conceivable that the dissipation process would take a few min to reach an “adequate” level. In other words, enhanced synchrony very likely plays a fundamental role in kindling, in terms of driving more electrophysiological and correlative structural events in the network. Additional extrinsic pacing before adequate dissipation of the previously aroused activities may perturb the ordered synchrony and thus have an anticonvulsant effect. The demarcation between the proconvulsant and anticonvulsant, or kindling and quenching effect, therefore is critically dependent on the level of dissipation of the triggered AD in the network. The DC pulses, which have no kindling effect (Figure 1) and trigger no activities but abolish the synchronized activities in the prelimbic cortex-medial thalamus-BLA networks (Supplementary Figures 4, 5, Medeiros and Márcio, 2014; Chou et al., 2020), therefore, might make an ideal anticonvulsant maneuver if given frequently enough to the seizure network (i.e., to be presented to the system assuredly before the dissipation of AD). This is probably part of the reason why brain stimulation shows rather variable outcomes in seizure treatment (Vonck et al., 2002; Wyckhuys et al., 2007; Bondallaz et al., 2013; Laxpati et al., 2014; Zhou et al., 2018). Given the higher probability of tissue injury with more frequent stimulation, we further demonstrated that 10 s *in situ* DCS given every 5 min (∼3% of the duty cycle) has a prominent quenching effect (Figures 4, 5). If such a protocol proves its efficacy when applied to another site in the seizure network (Figure 8) and its long term safety (Figure 9; Gaito, 1981; Weiss et al., 1998; Mina et al., 2015), it could be intermittently given on a simple open-loop basis which may be much more technically feasible than the complicated closed-loop design. Compared to ablation surgeries, intermittent *in situ* DCS could provide an access to difficult areas not feasible for resection, or to multiple epileptogenic foci with multiple electrodes (or even with just one electrode if these foci are resonating parts of the same seizure network). At the molecular level, continuous depolarization by DC currents (Bikson et al., 2004) for 10 s may drive many Na^+^ channels into the slow inactivated state, the recovery from which could take up to a few tens of seconds to minutes, leading to a prolonged change in neural excitability and jeopardizing synchrony. If so, it would be interesting to explore the collaborative effect of DCS of this kind and antiseizure drugs based on Na^+^ channel inhibition. Lacosamide probably is, especially, of interest in this regard, for its selective binding and stabilization of the slow inactivated state of Na^+^ channels (Errington et al., 2008; Wang et al., 2010; Peng et al., 2020).

## Data Availability Statement

The original contributions presented in the study are included in the article/Supplementary Material, further inquiries can be directed to the corresponding author/s.

## Ethics Statement

The animal study was reviewed and approved by Institutional Animal Care and Use Committee of National Taiwan University College of Medicine.

## Author Contributions

PC performed the experiments and analyzed the data. C-CK supervised the findings and the analysis of this work. Both the authors contributed to the final version of the manuscript.

## Conflict of Interest

The authors declare that the research was conducted in the absence of any commercial or financial relationships that could be construed as a potential conflict of interest.

## Publisher’s Note

All claims expressed in this article are solely those of the authors and do not necessarily represent those of their affiliated organizations, or those of the publisher, the editors and the reviewers. Any product that may be evaluated in this article, or claim that may be made by its manufacturer, is not guaranteed or endorsed by the publisher.
