# Supplementary material for: Anticonvulsant vs. Proconvulsant Effect of in situ Deep Brain Stimulation at the Epileptogenic Focus
Source: Front Syst Neurosci. 2021 Aug 2;15:607450. doi: 10.3389/fnsys.2021.607450 (PMC8366291; doi:10.3389/fnsys.2021.607450)
Supplement: Supplementary file 1 [file Data_Sheet_1.pdf]

Supplementary Material

1 Supplementary Figures

Supplementary Figure 1. No AD elicited with 1 Hz or DC stimulation

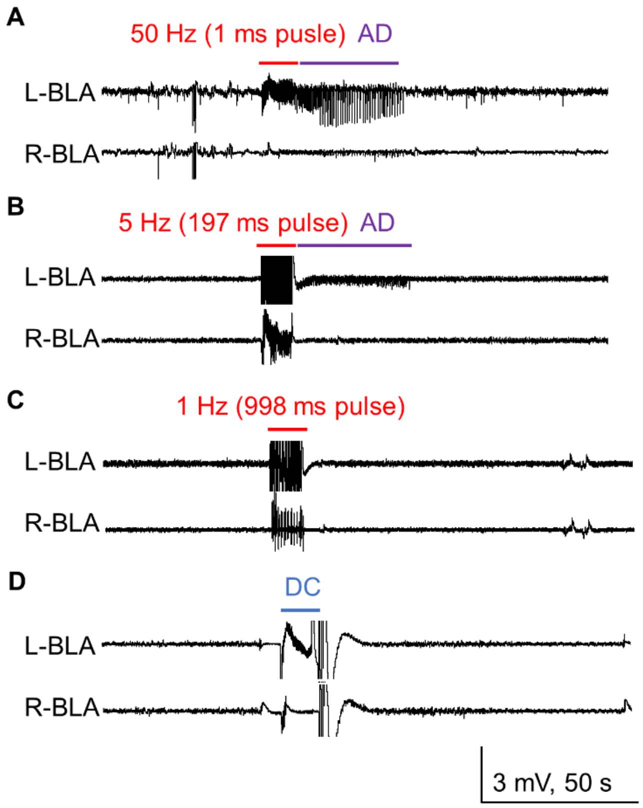

(A) to **(D)** Sample LFP recordings show that AD were elicited by 10-s 50 Hz (1 ms pulse width) or 5 Hz (197 ms pulse width) but not 1 Hz (998 ms pulse width) or DCS applied to left BLA. The stimulating currents were ± 120 μA, ± 220 μA, ± 300 μA, and 500 μA for 50 Hz, 5 Hz, 1 Hz, and DC, respectively.

Supplementary Figure 2. The dose-dependent curves of DC stimulation on kindling- or challenge-induced AD.

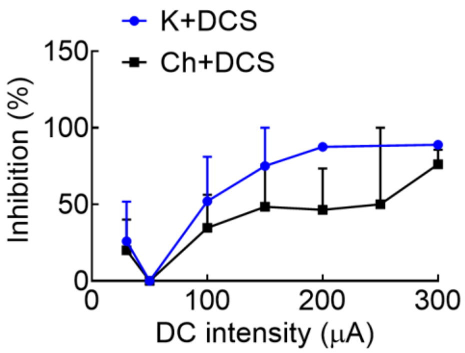

DC currents were applied for 10 s with the kindling ("K") or challenge ("Ch") stimuli. The current amplitude was set to 30 μA at first, and then increased to 50 μA, and then to 300 μA in a 50 μA increment. The percentage of inhibition is the ratio between the number of sessions in which AD is not present and the number of total sessions at each current. (n = 3 and 4 for kindling and challenge groups, respectively)

Supplementary Figure 3. Lack of apparent effect of DC stimulation on locomotor activities.

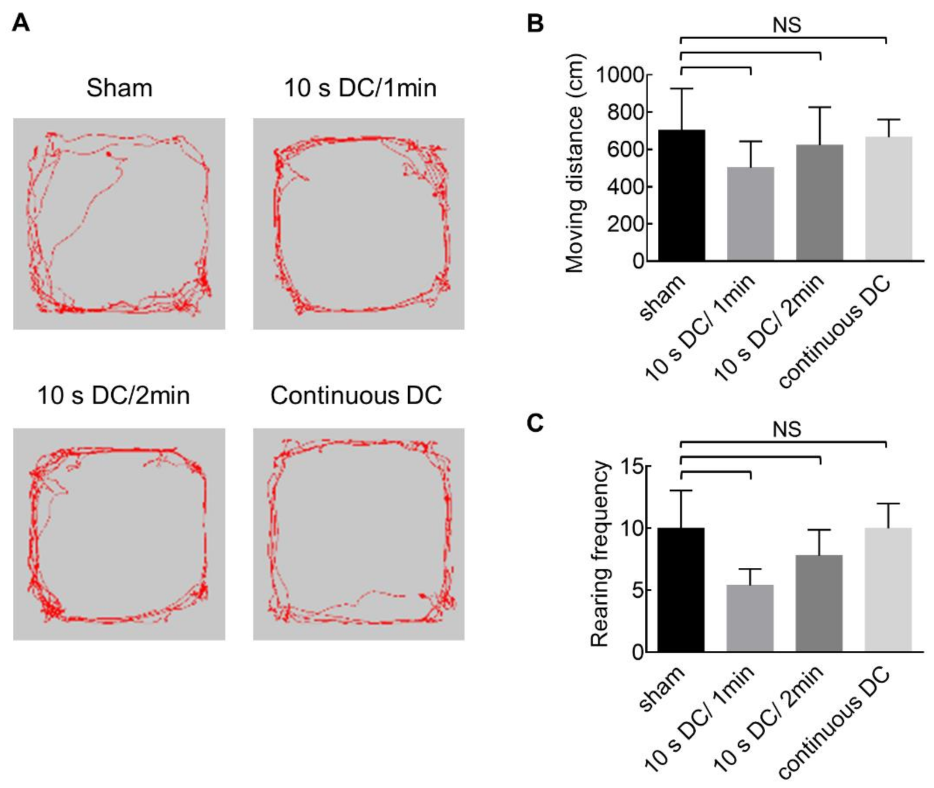

(A) Sample 5-minute free-running traces of a normal (unkindled) rat in an arena. DCS was not applied ("sham"), or DCS (100 μA x 10 s) was applied to left BLA every minute ("10 s DC/ 1 min") or every two minutes ("10 s DC/ 2 min"), or 100 μA DC DBS was continuously applied for the whole 5-min period ("continuous DC"). **(B)** and **(C)** Cumulative results in part A show no significant differences in total moving distance **(B)** and rearing frequency **(C)** in the 5-min period (n = 5) for the different conditions of DCS if compared to baseline ("sham"). NS: $p \geq 0.05$, Wilcoxon matched-pairs signed rank test.

Supplementary Figure 4. Lack of apparent changes in the power spectrums immediately before and after DC stimulation.

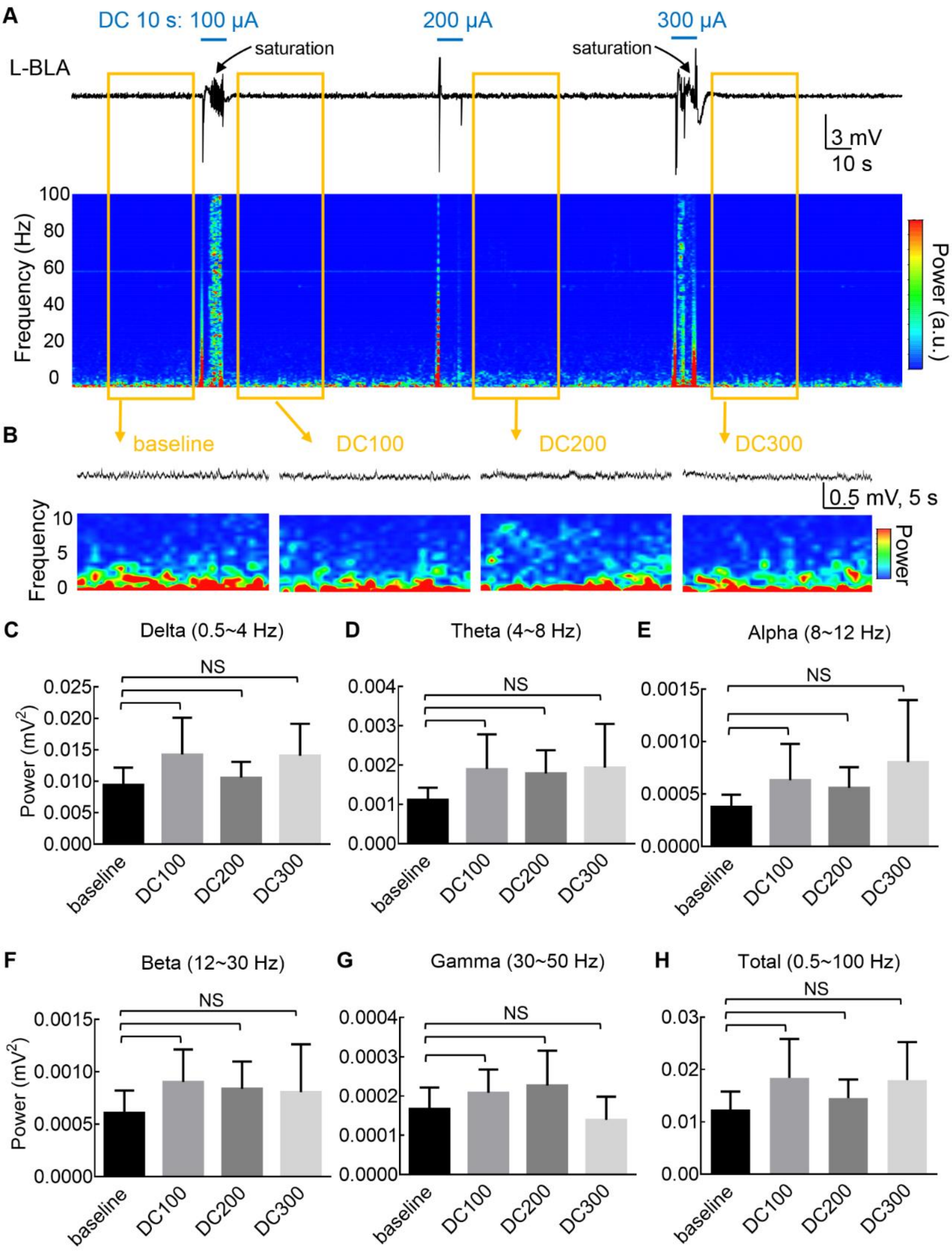

(A) Samples LFP (upper panels) and the spectrogram (lower panels) before and after DCS (100 μA, 200 μA, or 300 μA x 10 s) at left BLA are shown. **(B)** A closer view

of the boxed areas in part A (zoomed chiefly for the delta-theta frequency range which constitutes the predominant or "natural" rhythms in BLA, Chou et al., 2020) shows no apparent changes in the spectrograms before and after DCS, no matter the stimulating currents are 100 μA, 200 μA, or 300 μA. **(C-H)** The power of different frequency bands (from delta to gamma) and total power for the boxed areas in part A are documented, showing no significant changes after stimulation ("DC 100", "DC 200", and "DC 300") if compared to baseline (before stimulation). (n = 4). NS: $p \geq 0.05$, Wilcoxon matched-pairs signed rank test.

Supplementary Figure 5. Suppression of AD and delta synchronization after challenge in the network by DC stimulation at left BLA.

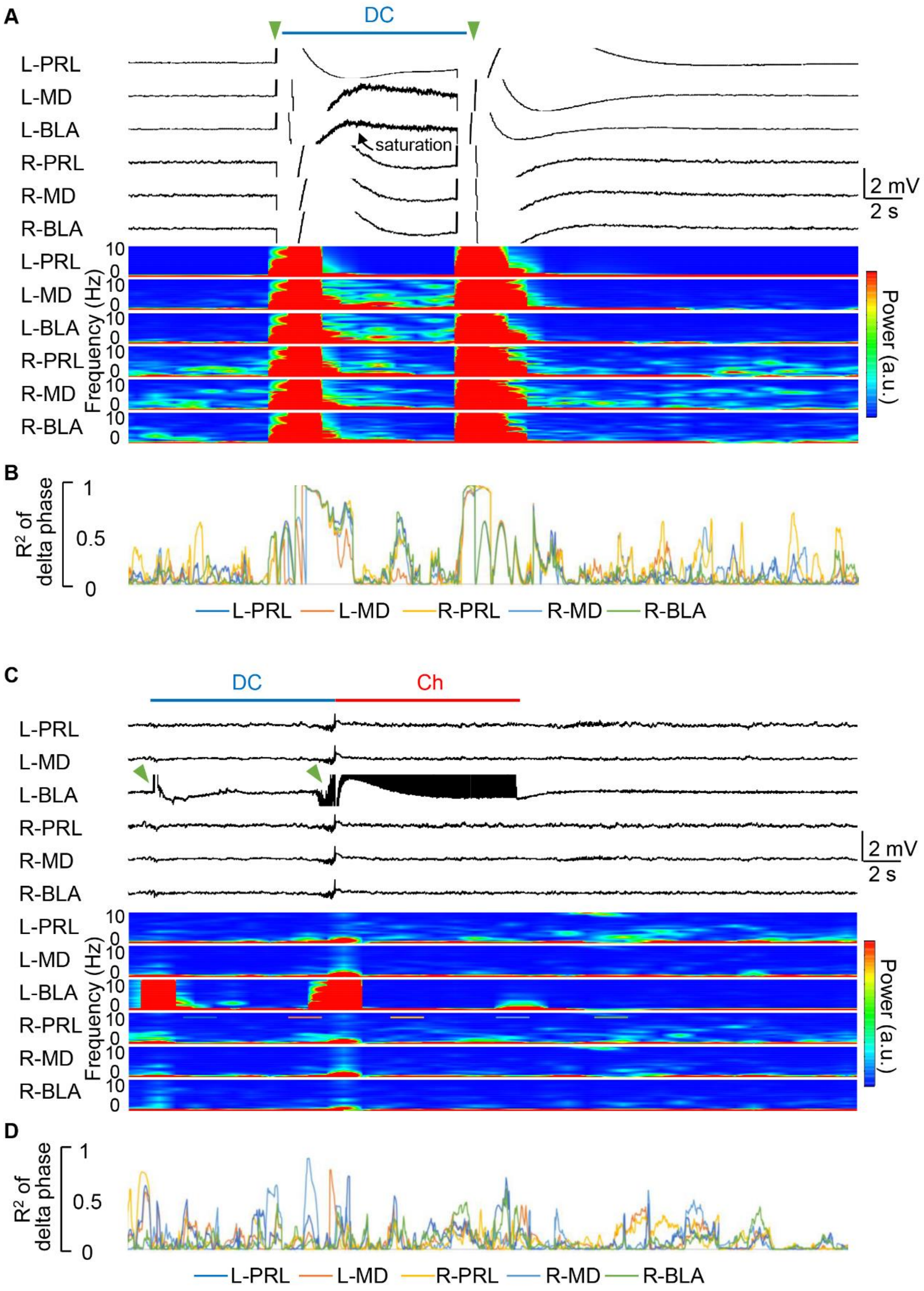

(A) Simultaneous LFP sweeps and spectrograms are obtained from bilateral BLA, thalamic mediodorsal nuclei (MD), and prelimbic cortices (PrL) before, during, and immediately after a 10-sec 100 μA DCS at left BLA (L-BLA). Signals in 6 areas remain similar before and after the DCS. The recording electrodes were sometimes saturated by DC stimulation, and the on-off stimulation artifacts are always prominent in both LFP sweeps and spectrograms (green arrows). **(B)** Phase synchronization of delta oscillations between left BLA and different structures in part A shows no synchrony event evoked after DC stimulation. **(C)** Sample LFP sweeps and spectrograms from different structures in part A show that DCS right before the challenge stimuli applied to left BLA markedly suppresses both elicited potentials in the other locations than left BLA during the challenge and AD in all locations after DC stimulation. **(D)** No phase synchronization of delta oscillations between left BLA and the other structures after the challenge if DCS was given beforehand.

2 Supplementary References

Chou, P., Wang, G.H., Hsueh, S.W., Yang, Y.C., & Kuo, C.C. (2020). Delta-frequency augmentation and synchronization in seizure discharges and telencephalic transmission. *iScience* 23(11), 101666. doi: 10.1016/j.isci.2020.101666
